# Comparison of the major cell populations among osteoarthritis, Kashin–Beck disease and healthy chondrocytes by single-cell RNA-seq analysis

**DOI:** 10.1038/s41419-021-03832-3

**Published:** 2021-05-27

**Authors:** Xi Wang, Yujie Ning, Pan Zhang, Blandine Poulet, Ruitian Huang, Yi Gong, Minhan Hu, Cheng Li, Rong Zhou, Mikko J. Lammi, Xiong Guo

**Affiliations:** 1 School of Public Health, Xi’an Jiaotong University Health Science Center, Key Laboratory of Trace Elements and Endemic Diseases, National Health and Family Planning Commission, 710061 Xi’an, Shaanxi P. R. China; 2 Institute of Lifecourse and Medical Sciences, Department of Musculoskeletal and Ageing Science, University of Liverpool, 6 West Derby Street, Liverpool, L7 8TX UK; 3 Shaanxi Provincial Institute for Endemic Disease Control, 710003 Xi’an, Shaanxi P. R. China; 4 Department of Integrative Medical Biology, University of Umeå, Umeå, Sweden

**Keywords:** Mechanisms of disease, Osteoarthritis

## Abstract

Chondrocytes are the key target cells of the cartilage degeneration that occurs in Kashin–Beck disease (KBD) and osteoarthritis (OA). However, the heterogeneity of articular cartilage cell types present in KBD and OA patients and healthy controls is still unknown, which has prevented the study of the pathophysiology of the mechanisms underlying the roles of different populations of chondrocytes in the processes leading to KBD and OA. Here, we aimed to identify the transcriptional programmes and all major cell populations in patients with KBD, patients with OA and healthy controls to identify the markers that discriminate among chondrocytes in these three groups. Single-cell RNA sequencing was performed to identify chondrocyte populations and their gene signatures in KBD, OA and healthy cells to investigate their differences as related to the pathogenetic mechanisms of these two osteochondral diseases. We performed immunohistochemistry and quantitative reverse-transcription PCR (qRT-PCR) assays to validate the markers for chondrocyte population. Ten clusters were labelled by cell type according to the expression of previously described markers, and one novel population was identified according to the expression of a new set of markers. The homeostatic and mitochondrial chondrocyte populations, which were identified by the expression of the unknown markers MT1X and MT2A and MT-ND1 and MT-ATP6, were markedly expanded in KBD. The regulatory chondrocyte population, identified by the expression of CHI3L1, was markedly expanded in OA. Our study allows us to better understand the heterogeneity of chondrocytes in KBD and OA and provides new evidence of differences in the pathogenetic mechanisms between these two diseases.

## Introduction

Kashin–Beck disease (KBD), an endemic and chronic degenerative osteochondral disease with irreversible pathological and clinical signs, is characterised with clinical manifestations including shortened and enlarged fingers at early stage of onset of KBD, and deformed limb joints, limited movement and even dwarfism in some patients with advanced disease^1,2^. Osteoarthritis (OA) is the most common chronic disease, involving progressive joint dysfunction with ageing. Several similar pathological changes have been found in KBD and OA, including extracellular matrix (ECM) degradation, cartilage lesions, and reduction and disruption of proteoglycans (PGs) and collagens^3–6^. However, the age of onset, symptoms and X-ray findings differ between KBD and OA^3^, which suggests that they are caused by different aetiologies and have distinct pathogeneses. Meanwhile, adult KBD patients typically have advanced KBD, often accompanied by OA^4,6^. Recent studies have found differences in various cellular pathways between KBD and OA chondrocytes^5,7,8^, suggesting differences in multiple processes, such as metabolism, apoptosis, adaptive immune defence, cytoskeleton, cell movement and extracellular matrix turnover^4,9–12^. However, these studies do not take into consideration the heterogeneity of articular cartilage cell types and therefore may be missing important information on cartilage degeneration processes specific to KBD or OA.

Articular cartilage is a highly organised structure composed of multiple zones, namely, the superficial, middle, deep and calcified zones, which display three chondrocyte phenotypes: persistent, transient and hypertrophic cells^13,14^. The chondrocyte phenotype, cell shape, and extracellular matrix (ECM) structure are different among the different zones^13–15^. In a previous study, seven defined populations of chondrocytes were identified in OA cartilage, specifically, proliferative chondrocytes (ProCs), prehypertrophic chondrocytes (PreHTCs), hypertrophic chondrocytes (HTCs), fibrocartilage chondrocytes (FCs) and three novel populations with distinct functions^16^. In addition, senescent cells (SNCs) and cartilage progenitor cells (CPCs) were also identified in recent studies^17–20^. Specific chondrocyte populations may play important roles in osteochondral diseases such as KBD and OA. For example, PreHTCs are capable of regulating the onset of hypertrophic differentiation, while HTCs can modulate the mineralisation of the surrounding matrix in the cartilage^16^, processes that are known to be more active in OA cartilage^21,22^. Additionally, CPCs have the ability to self-renew and differentiate along multiple lineages, thereby contributing to cartilage repair and homeostasis, and their dysregulation may affect and modulate cartilage loss in the processes of OA and KBD^17,20^.

One of the main differences in pathological changes between KBD and OA is in the location of the primary cartilage loss. OA is characterised primarily by progressive degradation starting in the superficial zone, with subsequent inflammation. In contrast, the primary characteristic pathological changes of KBD patients were chondrocyte necrosis occurring in the deep zone of articular cartilage and hypertrophic layers of epiphyseal plate cartilage^23,24^. However, the pathological changes in the different chondrocyte populations in KBD cartilage have not yet been studied, and we therefore do not yet have a full understanding of the difference in cartilage degeneration mechanisms between OA and KBD.

Single-cell RNA sequencing (scRNA-seq), a novel and powerful method for investigating transcriptomic cell-to-cell variation, is widely applied to identify various cell types and provide insights into the pathological processes of diseases^25–28^. In this study, scRNA-seq was used to better understand the changes occurring in the chondrocytes from patients with KBD and OA. The results allowed us to better understand the heterogeneity of chondrocytes between KBD and OA and provided us with new evidence to explore the differences in the pathogenetic mechanisms between these two diseases.

## Results

### scRNA-seq census of healthy human chondrocytes identified seven distinct cell populations

**Fig. 1:**
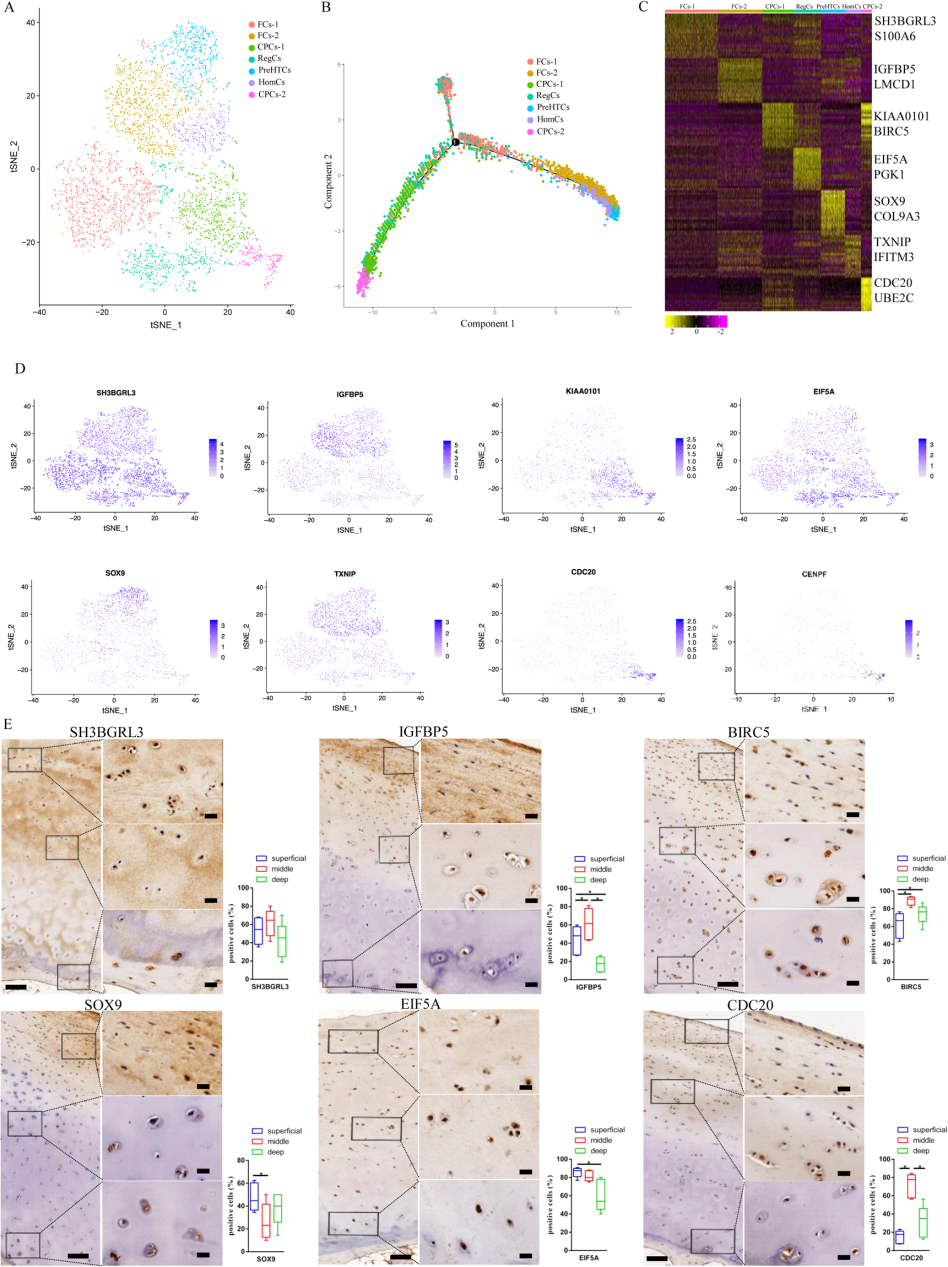
Single-cell RNA-sequencing analysis of human healthy control chondrocyte tissue samples. **A** Seven healthy human chondrocyte clusters. Visualisation of clustering by t-SNE plot of healthy control samples, identified by cell type. **B** Monocle pseudotime trajectory showing the progression of seven clusters of healthy chondrocytes. **C** Heat map of scaled gene expression data for the top 10 differentially expressed genes identifying each cluster, with selected genes listed. **D** Gene expression plots demonstrating high expression of SH3BGRL3 in SH3BGRL3^hi^ fibrocartilage chondrocytes-1, IGFBP5 in IGFBP5^hi^ fibrocartilage chondrocytes-2, CDC20 in CDC20^hi^ cartilage progenitor cells-1, KIAA0101 in KIAA0101^hi^ cartilage progenitor cells-2, EIF5A in EIF5A^hi^ regulatory chondrocytes, SOX9 in SOX9^hi^ prehypertrophic chondrocytes and TXNIP in TXNIP^hi^ homeostatic chondrocytes. Dot colour corresponds to the level of gene expression in each cell. t-SNE, t-distributed stochastic neighbour embedding. **E** Representative immunohistochemistry staining of SH3BGRL3, IGFBP5, BIRC5, SOX9, EIF5A and CDC20 in healthy cartilage tissues. Scale bar, left, 500 μm; right, 50 μm, and quantification of positive cells of different areas (superficial, middle, deep) in cartilage tissues displayed by box plot (*n* = 5). *p < 0.05.

To determine the cellular composition of chondrocytes, we profiled chondrocytes from healthy human cartilage tissues using scRNA-seq. Unbiased clustering of the chondrocytes resulted in seven clusters originating from healthy human cartilage tissues (Fig. 1A). Specifically, the following cells were identified: fibrocartilage chondrocytes-1 (FCs-1, expressing SH3BGRL3, S100A6 and MYL9), fibrocartilage chondrocytes-2 (FCs-2, expressing IGFBP5 and LMCD1), cartilage progenitor cells-1 (CPCs-1, expressing CDC20, UBE2C, CENPF and CKAP2), regulatory chondrocytes (RegCs, expressing EIF5A, PGK1, ANXA1 and TUBA1A), prehypertrophic chondrocytes (preHTCs, expressing SOX9, COL9A3 and COL11A1), homeostatic chondrocytes (HomCs, expressing TXNIP, IFITM3, GDF15 and TIMP1) and cartilage progenitor cells 2 (CPCs-2, expressing KIAA0101, BIRC5, CDKN3 and TMMN1) (Fig. 1C, D).

### Single-cell trajectory of normal chondrocyte differentiation

To study the differentiation of healthy human chondrocytes into different clusters and the corresponding gene expression, we used monocle to reconstruct the pseudotime trajectory (Fig. 1B). We found that the root of the trajectory was mainly populated by CPCs-1 and CPCs-2, while FC-1 was distributed in the middle of the trajectory, FC-2 was distributed along the trajectory, RegCs were located in front of FCs, and PreHTCs were behind FCs. PreHTCs and HTCs were mainly distributed at the end of the trajectory. The two termini of the tree were populated by FC-1 and RegCs for fate 1 and FC-2, PreHTCs and HomCs for fate 2 (Fig. 1B).

Next, we assessed the expression of genes whose levels varied during CPC differentiation in cells at fates 1 and 2 of the trajectory. The expression of COL1A1, IGFBP6, CALR, SULF1, ACTB, LGALS1 and PFN1 was found to be similar in both groups. COL1A1, IGFBP6, CALR and SULF1 expression was slightly reduced, while ACTB, LGALS1 and PFN1 expression was slightly increased, at the early stage of differentiation, notably downregulated in fate 2 cells and upregulated in fate 1 cells. FMOD, DDIT4, IGFBP5, BGN, ZFAS1, FTL, COMP and CHI3L1 were slightly downregulated at the early stage of differentiation, notably upregulated in fate 2 and downregulated in fate 1. In contrast, CEMIP, ACTA2, PTX3, TAGLN, CCL2, ADAMTS1 and SERPINE1 expression decreased slightly in cells from the root to fate 2 but markedly increased in cells that differentiated via fate 1 (Supplementary Fig. 1).

To study the distribution of different cell clusters, we used immunohistochemistry to detect marker gene expression (Fig. 1E). The results revealed that FCs were mainly distributed in the superficial and middle zones, CPCs were mainly distributed in the middle and deep zones, RegCs were mainly distributed in the superficial zone, and PreHTCs were mainly distributed in the superficial and deep zones.

### Systemic comparison of the single-cell landscape among KBD, OA and healthy human chondrocytes

**Fig. 2:**
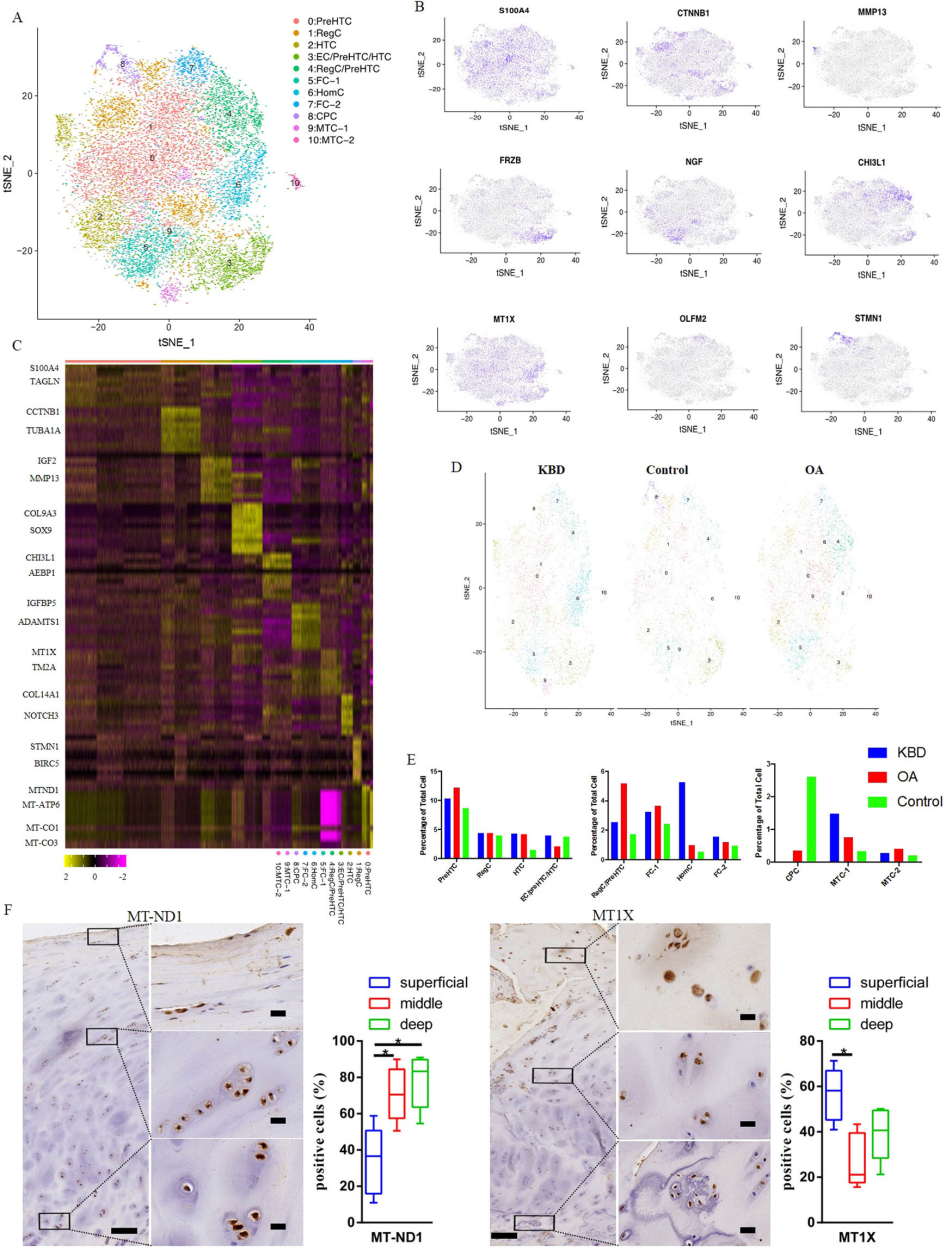
Single-cell RNA-sequencing analysis of human healthy control, KBD chondrocyte and OA chondrocyte tissue samples. **A** Visualisation of clustering by t-SNE plot of all combined healthy control, KBD and OA samples, identified by cell type. **B** T-SNE plots demonstrating the gene expression of previously described markers used to identify the cell identity of each cluster: S110A4-prehypertrophic chondrocytes; CTNNB1- regulatory chondrocytes type 1; MMP13- hypertrophic chondrocytes; FRZB- effector chondrocytes; CHI3L1-regulatory chondrocytes type 2; NGF-proliferative chondrocytes; MT1X-homeostatic chondrocytes; OLFM2-fibrocartilage chondrocytes; STMN1-cartilage progenitor cells. Dot colour corresponds to the gene expression level in each cell. t-SNE, t-distributed stochastic neighbour embedding. **C** Heat map of scaled gene expression data for the top 5 differentially expressed genes identifying each cluster, with selected genes listed. t-SNE, t-distributed stochastic neighbour embedding. **D** t-SNE plot of cells according to disease status; different clusters contained different numbers of cells from KBD, OA and control samples. **E** Mean percentage of total cells that comprised each cell type, comparing control, KBD and OA samples. Bars indicate the percentage of total cells. **F** Representative immunohistochemistry staining of MT-ND1 and MT1X in KBD cartilage tissues. Scale bar, left, 500 μm; right, 50 μm, and quantification of positive cells of different areas (superficial, middle, deep) in cartilage tissues displayed by box plot (*n* = 5). *p < 0.05.

We compared scRNA-seq among healthy chondrocytes, KBD chondrocytes and OA chondrocytes. In total, 16,375 cells were analysed, with 6140 cells from patients with KBD, 5834 cells from patients with OA and 4401 cells from healthy controls. Ten clusters were labelled by cell type using the expression of previously described markers, and one novel population, mitochondrial chondrocytes (MTCs), was identified by the expression of novel markers (Fig. 2A). PreHTCs, identified as positive for S100A4, COL9A3 and AKR1C1, were divided into three primary subgroups of 6 clusters: the first expressing S100A4, TAGLN and SH3BGRL3 (S100A4^hi^), the second expressing COL9A3, SOX9 and COL11A1 (COL9A3^hi^) and the third expressing AKR1C1, LUM and AKR1C2 (AKR1C1^hi^). RegCs were found in six separate clusters, which were divided into two primary subgroups: the first expressing CTNNB1, TUBA1A and CDC42 (CTNNB1^hi^) and the second expressing CHI3L1, AEBP1 and STEAP1 (CHI3L1^hi^). MTCs, the novel population in this study, were found in two separate clusters, the first expressing MT-ND1, MT-ATP6 and MT-ND2 (MT-ND1^hi^) and the second expressing MT-CO1, MT-CO3 and MT-CYB (MT-CO1^hi^). FCs were found in seven separate clusters, which were divided into two primary subgroups: the first expressing IGFBP5, TPM1 and ADAMTS1 (IGFBP5^hi^) and the second expressing COL14A1, NOTCH3 and COL6A3 (COL14A1^hi^) (Fig. 2B, C and Supplementary Fig. 2).

When separating t-SNE plots for each disease state, we noted that cluster 8, representing CPC cells, was present only in normal cartilage and was largely missing in both OA and KBD samples (Fig. 2D). In addition, an analysis of the proportion of total cells present in each population by sample and disease status revealed several significant changes among normal chondrocytes, KBD chondrocytes and OA chondrocytes (Fig. 2E). The populations of RegCs and prehypertrophic chondrocytes increased in OA chondrocytes compared with healthy control and KBD chondrocytes. Meanwhile, the numbers of hypertrophic chondrocytes, FCs and MTCs tended to increase from controls and OA to KBD.

### Expanded cell population cluster in KBD samples

Two distinct chondrocyte populations seem to be expanded mainly in KBD samples compared to normal and OA samples. These include the novel MTCs and HomCs (Fig. 2D). HomCs were identified by their expression of the known markers MT1X, MT2A, MT1E, ATF5 and DDIT3. Another distinct population that was markedly expanded in KBD was the MTCs, which is a newly defined population that is identified by a signature that includes genes related to mitochondrial electron transport and the response to hydroperoxide, including the MT-ND1, MT-ATP6, MT-ND2 and MT-ND3 genes. The expression of MT-ND1 and MT1X in cartilage tissues was investigated by using immunohistochemistry assays, which validated the existence of MTCs and HomCs in KBD cartilage. The results showed that MTCs were mainly distributed in the middle and deep zones, and HomCs were mainly distributed in the superficial zone in KBD cartilage (Fig. 2F). Moreover, we validated the gene expression of MT-ND1 and MT1X in patients with KBD using RT-PCR and found that the results were consistent with the single-cell RNA-seq analysis (Supplementary Fig. 3).

### Expanded cell population cluster in OA and normal samples

**Fig. 3:**
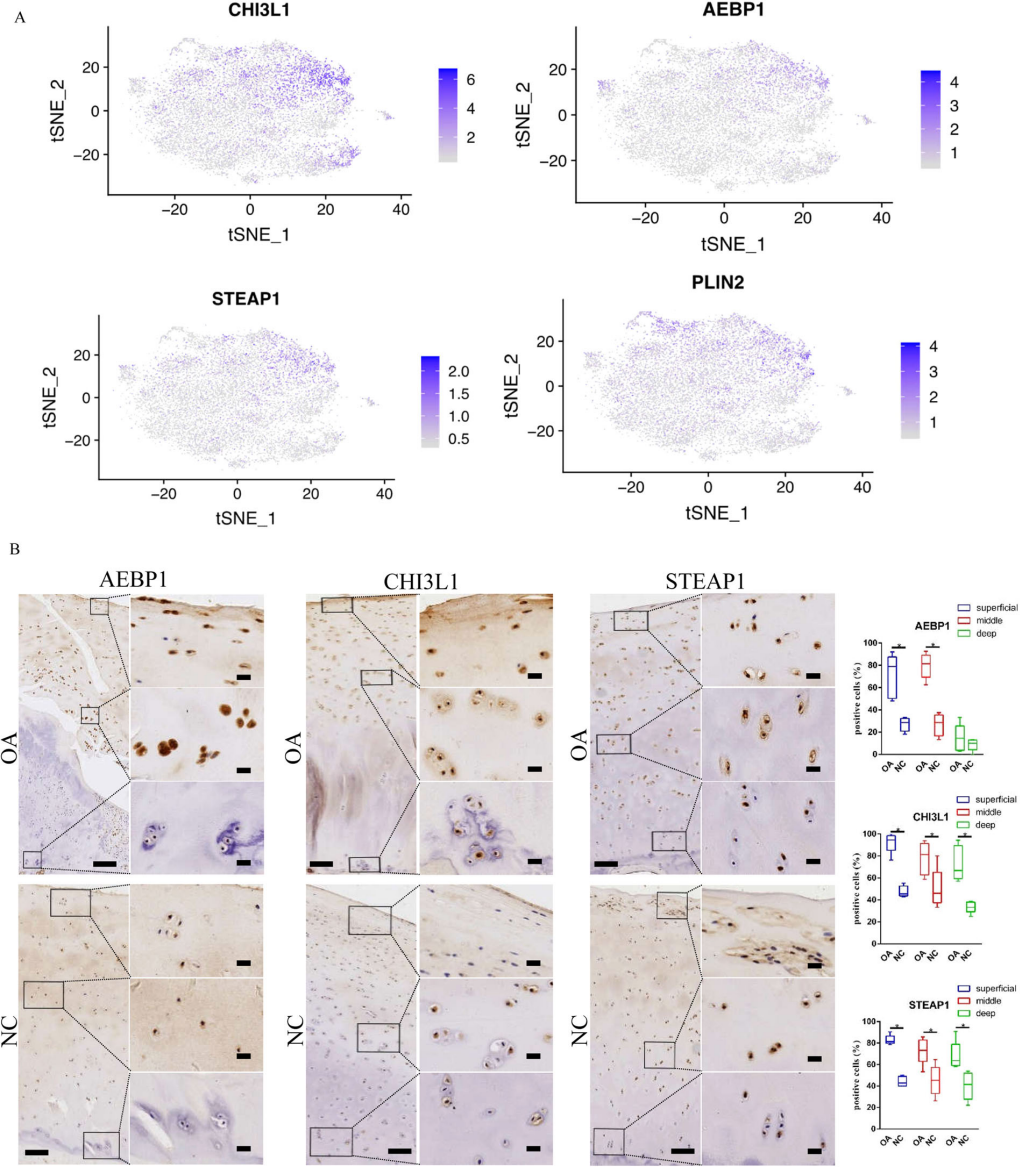
Single-cell RNA-sequencing analysis of the cell population in OA. **A** Gene expression plots of CHI3L1, AEBP1, STEAP1 and PLIN2. Dot colour corresponds to the level of gene expression in each cell. t-SNE, t-distributed stochastic neighbour embedding. **B** Representative immunohistochemistry staining of AEBP1, CHI3L1 and STEAP1 in OA and healthy cartilage tissues. Scale bar, left, 500 μm; right, 50 μm, and comparative quantification of positive cells of different areas (superficial, middle, deep) between OA and healthy cartilage tissues displayed by box plot (*n* = 5). *p < 0.05.

A distinct regulatory chondrocyte population that was markedly expanded in OA (Fig. 2D) was identified by its expression of the known markers CHI3L1, AEBP1, PLIN2 and STEAP1 (Fig. 3A). The expression of AEBP1, CHI3L1 and STEAP1 in cartilage tissues was investigated by using immunohistochemistry assays and validated the existence of RegCs in OA cartilage. The results showed that AEBP1 was upregulated in the superficial and middle zones, and CHI3L1 and STEAP1 were upregulated in the superficial, middle and deep zones of OA cartilage tissues compared to normal cartilage tissues (Fig. 3B). In addition, we validated the gene expression of AEBP1 and CHI3L1 in patients with OA using RT-PCR and found that the results were consistent with the single-cell RNA-seq analysis (Supplementary Fig. 3).

**Fig. 4:**
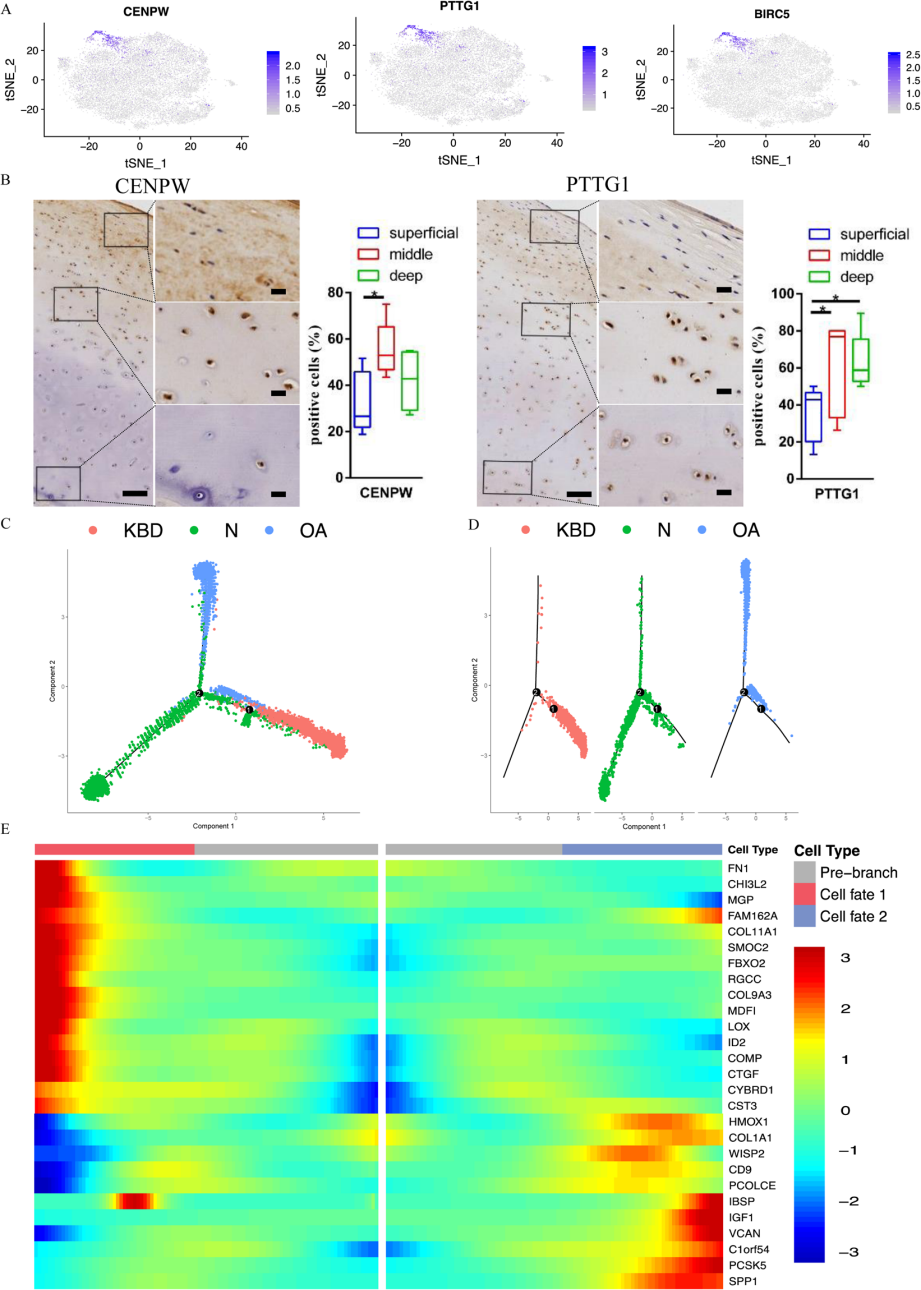
Single-cell RNA-sequencing analysis of the cell population in healthy controls. **A** Gene expression plots of CENPW, PTTG1 and BIRC5. Dot colour corresponds to the level of gene expression in each cell. t-SNE, t-distributed stochastic neighbour embedding. **B** Representative immunohistochemistry staining of CENPW and PTTG1 in healthy cartilage tissues. Scale bar, left, 500 μm; right, 50 μm, and quantification of positive cells of different areas (up, middle, deep) in healthy cartilage tissues displayed by box plot (*n* = 5). *p < 0.05. **C**, **D** Monocle pseudotime trajectory showing the progression of KBD, OA and the control. **E** The expression of the genes in a branch-dependent manner. Each row indicates the standardised kinetic curves of a gene. The centre of the heatmap shows the kinetic curve value at the root of the trajectory. From the centre to the left of the heatmap, the kinetic curve progresses from the root along the trajectory to fate 1. Starting from the right, the curve from the root to fate 2.

Distinct CPCs, which were markedly expanded in normal tissue (Fig. 2D), were identified by their expression of the known markers STMN1, PTTG1, CDKN3, BIRC5, UBE2S and CENPW (Fig. 4A). Although none of these markers were exclusive to CPCs, this cluster was the only population expressing all of these genes. We investigated the expression of CENPW and PTTG1 in cartilage tissues by using immunohistochemistry assays and validated the existence of CPCs in normal cartilage (Fig. 4B). The results revealed that CENPW was upregulated in the middle zone and PTTG1 was upregulated in the middle and deep zones in cartilage tissues from normal controls. In addition, the expression of PTTG1 and BIRC5 was investigated in cartilage tissues from OA and KBD patients, and the results showed that PPTG1 and BIRC5 were upregulated in the superficial zone of OA cartilage, while they were upregulated in the deep zone of KBD cartilage (Supplementary Fig. 4).

### Alignment of single-cell trajectory in normal, KBD and OA chondrocytes

To further investigate the relationship between the different disease statuses, we used monocle to study the transitions across specific cell types and sample states. We found that the pseudospace trajectory axis derived from monocle yielded three different trajectory states (Fig. 4C). Chondrocytes from normal cartilage were present at the start of the pseudospace trajectory, which then branched into 2 distinct fates, populated mainly with either KBD (fate 1) or OA (mostly fate 2) (Fig. 4D).

Next, we assessed the expression of genes regulated during chondrocyte differentiation in cells at fates 1 and 2 of the trajectory. The expression levels of FN1, CHI3L2, RGCC, COL9A3 and MDFI were markedly upregulated in cells at fate 1, whereas those of COL11A1, SMOC2 and FBXO2 were slightly downregulated at the early stage of differentiation and notably upregulated in cells at fate 1. HMOX1, COL1A1, WISP2, CD9 and PCOLCE were notably upregulated in cells at fate 2 and downregulated in cells at fate 1. IGF1, VCAN, PCSK5 and SPP1 were upregulated only in cells at fate 2 (Fig. 4E). These genes were differentially expressed at the prebranch point, suggesting that these genes may play a critical role in regulating the phenotype of chondrocytes at the beginning of the process of differentiation or development of chondrocytes from healthy controls to KBD or OA.

### Prehypertrophic chondrocytes, hypertrophic chondrocytes, regulatory chondrocytes and effector chondrocytes

**Fig. 5:**
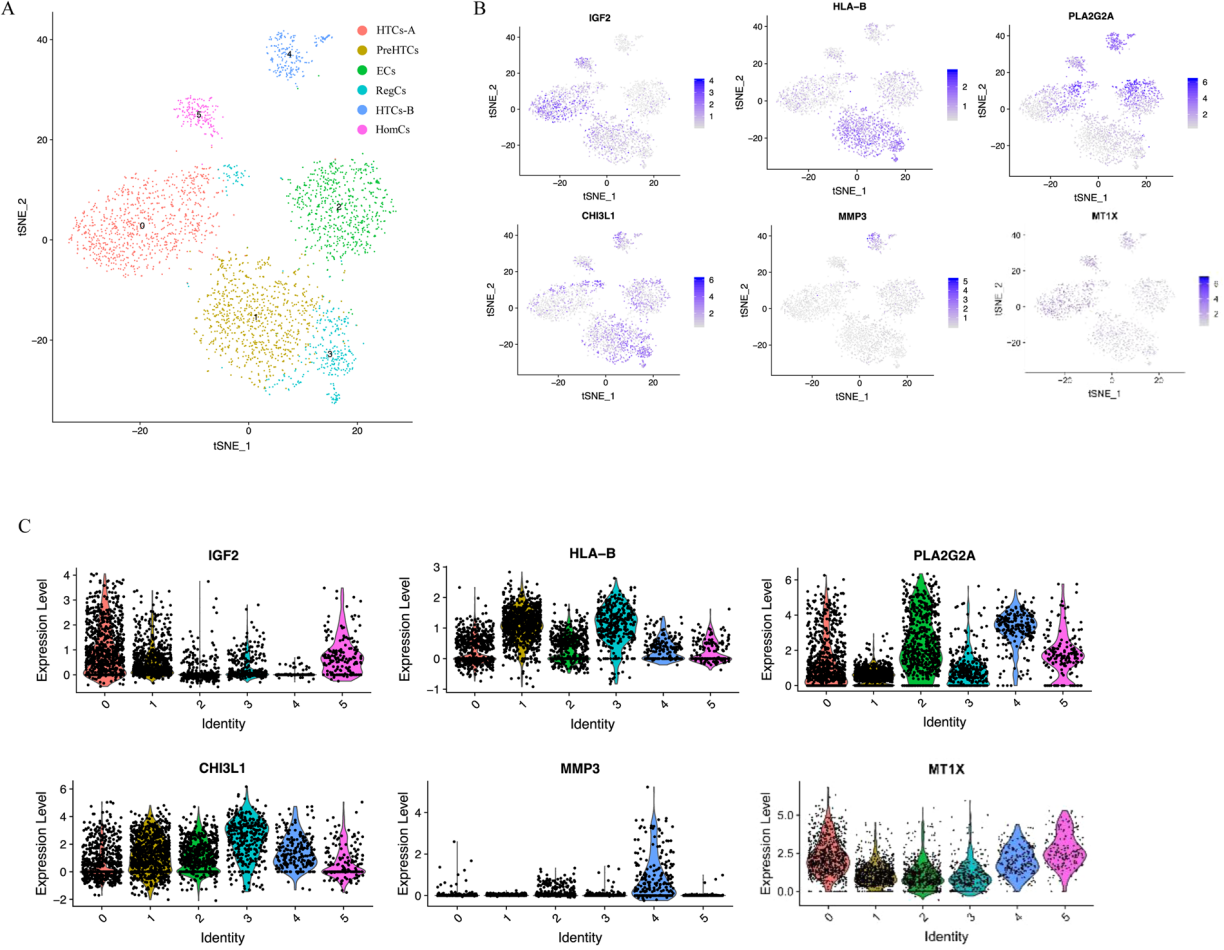
Recluster analysis of prehypertrophic chondrocytes, hypertrophic chondrocytes, regulatory chondrocytes and effector chondrocytes. **A** Visualisation of reclustering by t-SNE plot of the original cluster 3 and 4, identified by cell type. **B** T-SNE plots demonstrating the gene expression of markers used to identify the cell identity of each cluster. Dot colour corresponds to the level of gene expression in each cell. **C** Violin plots of gene expression of chondrocyte markers by reclustering original clusters 3 and 4.

Prehypertrophic chondrocytes, hypertrophic chondrocytes, RegCs and effector chondrocytes from clusters 3 and 4 (Fig. 5A, B) were combined and reclustered to allow clearer identification of these subpopulations. Hypertrophic chondrocytes-A (HTC-A) and hypertrophic chondrocytes-B (HTC-B) were marked by the robust expression of IGF2 and SLC5A3, MMP3 and GDF5. PreHTCs and ECs highly expressed S100A4 and HLA-B, MIA and PLA2G2A, respectively. CHI3L1 and AEBP1 were highly expressed in RegCs (Fig. 5C).

### Fibrocartilage chondrocyte subpopulation

**Fig. 6:**
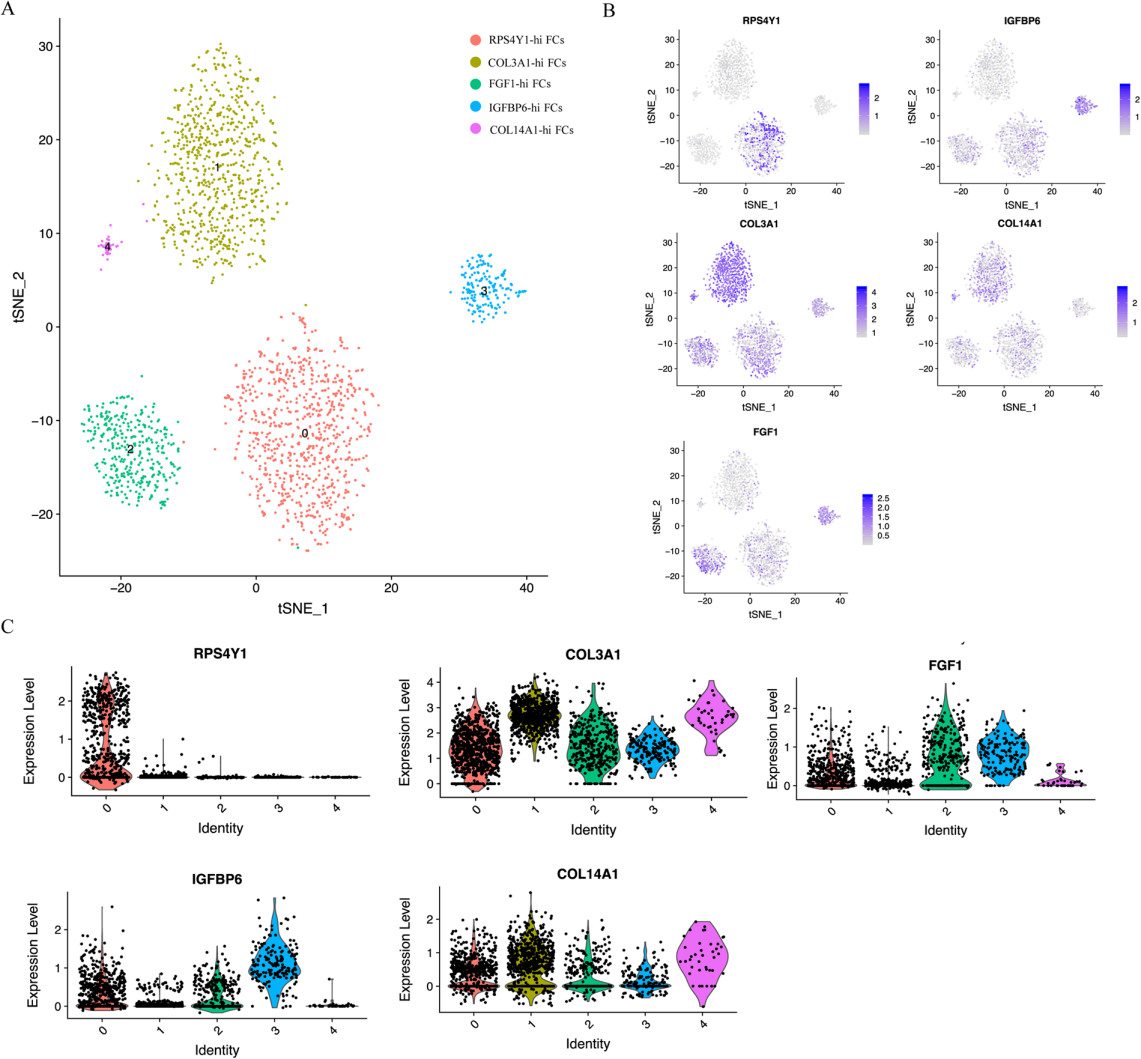
Recluster analysis of fibrocartilage chondrocytes. **A** Visualisation of reclustering by t-SNE plot of the original clusters 3 and 4, identified by cell type. **B** T-SNE plots demonstrating gene expression of markers used to identify the cell identity of each cluster. Dot colour corresponds to the level of gene expression in each cell. **C** Violin plots of gene expression of chondrocyte markers by reclustering original fibrocartilage chondrocytes.

Upon examining FC populations in the combined analysis of KBD, OA and healthy human chondrocytes, we again identified distinct populations of IGFBP5^hi^ and COL14A1^hi^ FCs, each containing KBD, OA and healthy human chondrocytes. Two major and three minor subpopulations of FCs emerged (Fig. 6A, B). The first major population was defined by the expression of RPS4Y1 and PENK (RPS4Y1^hi^ FCs). A second major population was defined by the expression of COL3A1 (COL3A1^hi^ FCs). Three minor populations were distinguished by the expression of FGF1, IGFBP6 and COL14A1 (Fig. 6C).

### Identification of effector and hypertrophic chondrocyte populations in KBD and OA chondrocytes

We separately analysed chondrocytes from patients with KBD and OA. Seven populations of chondrocytes were identified in both KBD and OA. The EC population was defined by the expression of MIA, S100B, FRZB, C2orf82 and S100A1 in both KBD and OA (Supplementary Fig. 5), suggesting that these markers from ECs were characterised as having the same expression pattern in KBD and OA. In addition, HTCs were defined by the expression of IBSP, MMP13, RUNX2 and TMEM119 in KBD chondrocytes (Supplementary Fig. 6).

## Discussion

The pathogenesis of KBD and OA is different in many aspects. KBD mainly invades the epiphyseal cartilage, epiphyseal plate cartilage and articular cartilage and occurs in children aged 3–12 years. This progressive lesion seriously affects the growth and development of children’s bones. Unlike KBD, OA is a degenerative chronic joint disease that mainly occurs in elderly individuals. Currently, we cannot distinguish KBD and OA well at the molecular level or propose precise treatment strategies for the two diseases. There are limited specific cell markers to report the internal status of chondrocytes in KBD and OA pathogenesis. Therefore, in this study, we performed a comprehensive analysis of chondrocyte populations in KBD, OA and healthy control cartilage. We identified a new population of chondrocytes, MTCs, and, identified the relationships among KBD, OA and healthy control chondrocytes at the cellular level. The HomC and MTC populations were markedly expanded in KBD. The RegC population was markedly expanded in OA, and CPCs were markedly expanded in healthy controls. Our results thus identified the major cell populations in patients with KBD, OA and healthy controls and the corresponding marker genes, which provide a novel foundation for further exploration of the differences in the pathogenetic mechanisms involved in KBD and OA.

Currently, mitochondrial studies are involved in biomedicine due to their critical roles in ageing and disease development in humans^29^. Mitochondria are organelles that can generate most of the energy essential to the cell by converting nutritional molecules into adenosine triphosphate (ATP). An increase the production of reactive oxygen species (ROS) and a decrease in the consumption of ATP and oxygen by a series of metabolic alterations can be caused by mitochondrial dysfunction. Mitochondrial dysfunction also leads to an inflammatory response, which induces the synthesis of cytokines and matrix metalloproteinases (MMPs)^30^. Compared with that in normal cartilage, mitochondrial biogenesis in KBD and OA chondrocytes is altered, such that there is a reduction in the activities of complexes II, III, IV and V^31,32^, which causes failure in energy generation. Hence, mitochondrial function needs correction to prevent excessive production of ROS and oxidative stress^30^. In this study, we identified a new population of MTCs that are markedly expanded in KBD and express genes related to ATP synthase in chondrocytes, the inflammatory response, calcium metabolism and chondrocyte survival^32^. These observations suggest that MTCs are active in energy supply. In a previous study, signs of mitochondrial dysfunction were identified in cultured KBD chondrocytes, including decreased cellular ATP levels, an increased ratio of cells with de-energised mitochondria, release of mitochondrial cytochrome c and elevated activation of caspases 9 and 3. In addition, the number of apoptosis-positive chondrocytes in patients with KBD was larger than that in healthy controls^24,31^. All these findings suggest that mitochondrial dysfunction and mitochondria-mediated cell death play critical roles in the pathophysiology of KBD.

We also identified a second novel cell population, HomCs. These cells were mainly present among KBD chondrocytes and exhibited high expression levels of metallothionein (MT) genes. MTs play diverse roles in metal detoxification, antioxidant functions, immune defence and cell differentiation. MTs are also important in regulating intracellular ROS levels^33,34^. A previous study also indicated that oxidative stress is the common pathological change in juvenile KBD patients recruited from both severely affected and mildly affected endemic regions. Severe oxidative stress is also associated with the occurrence and development of KBD^35^. The evidence above suggests that these HomCs might play a protective role against apoptosis by scavenging ROS and controlling cellular oxidative stress in KBD.

Although lacking intrinsic reparative ability, articular cartilage contains a population of cells with progenitor-like qualities^36–41^ that are similar to stem cells.^42^. This population of chondrocytes is known as CPCs and has been observed and isolated in human articular cartilage and characterised by their capacity for self-renewal, expression of stem cell-related surface markers and ability to differentiate along multiple lineages^17^. Since Dowthwaite et al.^43^ first reported the isolation of CPCs from the surface zone of articular cartilage, CPCs have been detected mostly in the superficial zone of articular cartilage in a number of studies^44,45^. CPCs have also been found to participate in cartilage self-repair during early or later stages of human OA^20,46,47^ and were found to be highly migratory towards damaged cartilage tissue and repopulated in repair tissue. However, another study indicated that PRG4-expressing cells served as a progenitor population of cartilage and were mainly found in superficial chondrocytes in young mice but expanded into deeper regions of articular cartilage as animals aged^48^. Yu et al.^49^ demonstrated that CPCs can be isolated from both the superficial and deep bovine articular cartilage and identified CPCs in healthy articular cartilage by a single-cell clonogenicity screening technique for the first time. In our study, the CPC population was mainly found in the middle and deep zones of normal healthy cartilage and possessed a high proportion of genes related to self-renewal ability, differentiation multipotency and migration^20,50^. The expression of CPC markers (PTTG1 and BIRC5) in OA and KBD cartilage tissues showed that CPCs were distributed in the superficial zone in OA cartilage and in the deep zone in KBD cartilage. These inconsistent results for the expression of CPC markers in OA and KBD may be caused by their different injury patterns to cartilage; OA is characterised by progressive degradation on the cartilage surface, while the degenerative changes in KBD cartilage are characterised by multiple foci of chondronecroses in the deep zone of the cartilage^24^. On the basis of these data, we inferred that the CPCs from the superficial zone in OA might actually originate or migrate from the middle and deep zones of CPCs in healthy articular cartilage. Moreover, CPCs could migrate extensively towards damaged cartilage tissue parts and repopulate in repair tissue.

Because healthy controls and KBD patients now rarely undergo surgical cartilage biopsy, our study was limited by a relatively small sample size. Cartilage samples were collected from only three pairs of patients and healthy controls, which may lead to statistical bias caused by the individual characteristics of the limited number of subjects. In addition, there is no evidence of sex differences in KBD, but a large number of studies have shown that women have a higher prevalence of knee OA than men, particularly after the age of 50^51,52^. In this study, to avoid the statistical bias caused by sex differences, all three cartilage samples used in scRNA-seq analysis were collected from female donors; however, we cannot ignore the influence of the sex bias of knee OA on the outcomes of the scRNA-seq analysis in future analyses. Therefore, we performed IHC to verify the results of scRNA-seq analysis and confirm similar results in cartilage from both women and men.

In conclusion, our study systemically compared the single-cell landscapes of KBD, OA and healthy chondrocytes. This analysis identified discriminative markers for specific chondrocyte populations, which may be considered targets for cartilage injury repair or key molecules in the pathogeneses of these two diseases. A new population, MTCs, was markedly expanded in KBD, and this expansion may represent an important mechanism of chondrocyte degeneration in KBD.

## Materials and methods

### Sample recruitment

The patients with KBD were diagnosed strictly according to the national diagnostic criteria of KBD in China [WS/T 207-2010]. The patients with OA were diagnosed strictly according to the Modified Outerbridge Classification. All subjects with alterations such as defects and sclerosis on the bone end of phalanges combined with compression changes of the calcaneus and talus and enlarged/deformed limb joints (hand, elbow, knee, ankle, etc.) manifested on X-ray were diagnosed with KBD. Subjects were excluded if they were suffering or had previously suffered from any other osteoarticular diseases (such as rheumatoid arthritis, gout or skeletal fluorosis) or any other type of macrosomia, disorder of osteochondrodysplasia or chronic disease (such as hypertension, diabetes or coronary heart disease) or had received any treatment in the past six months. Clinical information was collected from patient records. Articular cartilage samples from KBD and OA patients were collected from individuals who underwent arthroplasty of the knee. Healthy controls were obtained from the patients who suffered trauma or amputation due to an accident.

All donors signed a written informed consent form. All subjects were of Chinese Han lineage. Adult articular cartilage specimens were collected from one KBD patient, one OA patient and one healthy subject (Table S1) for single-cell RNA-seq and from five KBD patients, five OA patients and five healthy subjects (Table S2) for immunohistochemistry verification and from three KBD patients, three OA patients and three healthy subjects (Table S2) for qRT-PCR verification.

### Cartilage tissue collection and chondrocyte isolation

All articular cartilage samples, including all of the cartilage zones (including calcified) and subchondral bone, were harvested from the lateral tibial plateau and obtained within 1 h after operation. Chondrocytes were isolated as follows:^53–56^ articular cartilage specimens were washed twice with sterile phosphate-buffered saline (PBS) with antibiotics (penicillin and streptomycin), cut into pieces (1 mm^3^), and subjected to enzymatic digestion with 0.25% trypsin at 37 °C in an atmosphere of 5% CO_2_ for up to 30 min. Cell suspensions were centrifuged at 1000 × *g* for 5 min, the supernatant was aspirated completely, and the cells were digested in basal media supplemented with 0.2% type II collagenase at 37 °C using an Eppendorf Thermomixer for 12–16 h. Isolated chondrocytes were filtered through 70 mM nylon filters, washed twice with sterile PBS and then directly prepared for cDNA amplification and scRNA-Seq library construction.

### Single-cell RNA sequencing

#### Cell capture and cDNA synthesis

The Chromium Single Cell 3′ Library and Gel Bead Kit V2 (10x Genomics, 120237) and Single Cell A Chip Kit (10x Genomics, 120236) were used for cell capture. The cell suspension (300–600 living cells per microlitre, as determined by Count Star) was loaded onto the Chromium Single Cell Controller (10x Genomics) to generate single-cell gel beads in emulsion (GEMs) according to the manufacturer’s protocol. In short, single cells were suspended in PBS containing 0.04% BSA. Approximately 3173 cells were added to each channel, and the target cell recovery was estimated to be ~16,544 cells in total. Captured cells were lysed, and the released RNA was barcoded through reverse transcription in individual GEMs. Reverse transcription was performed on a S1000TM Touch Thermal Cycler (Bio Rad) at 53 °C for 45 min, followed by 85 °C for 5 min and a hold at 4 °C. cDNA was generated and then amplified, and quality was assessed using an Agilent 4200 (performed by CapitalBio Technology, Beijing).

#### Single-cell RNA-Seq library preparation

According to the manufacturer’s instructions, single-cell RNA-seq libraries were constructed using the Single Cell 3′ Library Gel Bead Kit V2. The libraries were sequenced using an Illumina NovaSeq6000 sequencer with a sequencing depth of at least 57,374 reads per cell with a paired-end 150 bp (PE150) reading strategy (performed by CapitalBio Technology, Beijing).

#### Seurat pipeline

Clustering was also performed with Seurat 3.0 (R package). Cells with a gene number was <200, a gene number ranked in the top 1%, or a mitochondrial gene ratio of >25% were regarded as abnormal and filtered out. Dimensionality reduction was performed using principal component analysis (PCA), and visualisation was realised by TSNE and UMAP.

#### Enrichment analysis

GO enrichment, KEGG enrichment, Reactome enrichment and disease enrichment (human only) of cluster markers were performed using KOBAS software with Benjamini–Hochberg multiple testing adjustment, using the top 20 marker genes for each cluster. The results were visualised in R.

#### Single-cell trajectory analysis

Single-cell trajectories were built with Monocle (R package) for pseudotime analyses. Genes were filtered by the following criteria: expressed in more than 10 cells; average expression value >0.1; and Qval < 0.01 in different analyses.

#### WGCNA

Weighted correlation network analysis (WGCNA) was performed by the WGCNA R software package with default parameters. According to the above clustering results, every cluster was divided into subclusters, and the average expression of genes in a subcluster was calculated.

#### Cell type annotation

Cell type annotation was carried out by SingleR (https://bioconductor.org/packages/devel/bioc/html/SingleR.html), which performs unbiased cell type recognition from single-cell RNA sequencing data by leveraging reference transcriptomic datasets of pure cell types to infer the cell of origin of each single cell independently. The human blueprint_encode and Human Primary Cell Atlas (HPCA) reference datasets were used.

### RNA extraction and quantitative reverse-transcription PCR verification

Total RNA was isolated from chondrocytes using the Trizol protocol. The RNA was then converted into complementary DNA (cDNA) by RT-PCR using the RevertAid™ First Strand cDNA Synthesis Kit (Thermo Scientific Molecular Biology, Canada) according to the manufacturer’s instructions. qRT-PCR was performed using an ABI7500 Real-Time PCR system (Applied Biosystems, Foster City, CA, USA) according to the manufacturer’s instructions. All primer and probe sets were supplied by TaqMan® Gene Expression Assays (Applied Biosystems). The relative expression level of the gene was calculated using the comparative Ct method. β-actin was used as an internal control to normalise the sample differences.

### Immunohistochemical verification

Cartilage tissues were fixed with 4% (w/v) paraformaldehyde for 24 h immediately after acquisition and decalcified in 10% (w/v) ethylenediaminetetraacetic acid disodium salt (EDTA-Na_2_) for 2–3 weeks. The samples were dehydrated in an alcohol series, cleared in xylene and embedded in paraffin wax. Paraffin sections were cut into 5-µm sections, mounted on slides and stored at room temperature until use for staining. The paraffin-embedded sections were baked at 65 °C for 1 h, deparaffinized with xylene and then rehydrated in decreasing concentrations of ethanol. Endogenous peroxidase activity was blocked by 0.3% (w/v) hydrogen peroxide for 10 min at room temperature, and then the sections were washed with 1×PBS. Then, the sections were incubated in 10 mol/L urea solution diluted with water at 37 °C for 20 min and washed with 1×PBS. For analysis of extracellular markers, such as IGFBP5 and CHI3L1, the sections were treated with 2 mg/ml hyaluronidase at pH 5.0 and 37 °C for 30 min. For analysis of intracellular markers, the sections were incubated in 0.1% trypsin/CaCl_2_ at 37 °C and digested for another 20 min for antigen retrieval. After blocking with 5% (w/v) goat serum for 20 min at room temperature, anti-SH3BGRL3 (1:200 dilution, bs-21171R, Bioss), anti-Survivin (1:100 dilution, bs-0615R, Bioss), anti-IGFBP5 (1:100 dilution, bs-0406R, Bioss), anti-SOX9 (1:100 dilution, bs-4177R, Bioss), anti-EIF5A (1:100 dilution, DF6754, Affinity Biosciences), anti-CDC20 (1:50 dilution, 10252-1-AP, Proteintech), anti-MT-ND1 (1:50 dilution, 19703-1-AP, Proteintech), anti-MT1X (1:50 dilution, 17172-1-AP, Proteintech), anti-AEBP1 (1:100 dilution, bs-0322R, Bioss), anti-CHI3L1 (1:100 dilution, bs-1093R, Bioss), anti-STEAP1 (1:100 dilution, bs-1901R, Bioss), anti-CENPW (1:100 dilution, bs-15230R, Bioss) or anti-PTTG1 (1:100 dilution, AF0354, Affinity Biosciences) antibodies and IgG as a negative control were applied on the sections, and the samples were further incubated overnight at 4 °C. After washing with 1×PBS, sections were incubated using a human serum amyloid P (SAP) kit (solution B contains a goat anti-rabbit secondary antibody; Zhongshan, Jinqiao, Guangzhou, China) according to the manufacturer’s instructions. The substrate 3,3′-diaminobenzidine was added to stain the sections, and haematoxylin counterstaining was performed. Finally, the sections were dehydrated and mounted under alcohol-cleaned coverslips. All IHC staining was assessed under light microscopy by two pathologists who were blinded to the origin of the samples. Each zone was identified based on previously reported characteristics^39,57–59^ and articular cartilage was divided into three cell morphologies, namely, the superficial, middle and deep zones, according to light microscopy observation. Chondrocytes in the superficial zone were relatively small and flat and were oriented with the long axis parallel to the surface; cells in the middle zone were larger and more rounded and were randomly distributed in the matrix with fibres running in oblique directions; and cells in the deep zone were larger in size and were arranged in a columnar manner perpendicular to the surface. Assessment of the staining throughout each cartilage zone included systematic counting of positive and negative cells, starting from the cartilage surface and progressing down through all layers of cartilage. Five randomly chosen fields in each zone were counted at ×50 magnification. The percentage of positive cells was calculated using the number of positively stained cells divided by the total number of cells (positively and negatively stained cells) in the chosen fields of view. The percentages of positive cells in different zones were calculated for each case and then for the different groups.

### Statistical analysis

Statistical analysis was performed using the SPSS 18.0 package. Differences in means were evaluated by one-way analysis of variance (ANOVA) with Tukey’s post hoc test for multiple comparisons. Student’s *t* test was applied to evaluate the difference between two groups. A normality test was applied first for continuous variables before any further comparison analyses. Nonparametric methods (e.g., Mann–Whitney U for pairwise comparisons) were used when the data were not normally distributed. A *P* value < 0.05 was considered to suggest a significant difference. The results of IHC and qRT-PCR are presented in bar charts using GraphPad PRISM 6 (GraphPad, San Diego, CA, USA).

## Supplementary information

Table S1

Table S2

Supplementary figure legends

online supplementary figure 1

online supplementary figure 2

online supplementary figure 3

online supplementary figure 4

online supplementary figure 5

online supplementary figure 6

## Data Availability

The datasets used and/or analysed during the current study are available from the corresponding author on reasonable request.

## References

[1] Ning Y (2018). Imbalance of dietary nutrients and the associated differentially expressed genes and pathways may play important roles in juvenile Kashin-Beck disease. J. Trace Elem. Med. Biol..

[2] Ning Y (2018). Dietary exosome-miR-23b may be a novel therapeutic measure for preventing Kashin-Beck disease. Exp. Ther. Med..

[3] Li Y (2013). Clinical features of Kashin-Beck disease in adults younger than 50 years of age during a low incidence period: severe elbow and knee lesions. Clin. Rheumatol..

[4] Li SY, Cao JL, Caterson B, Hughes CE (2012). Proteoglycan metabolism, cell death and Kashin-Beck Disease. Glycoconj. J..

[5] Duan C (2010). Comparative analysis of gene expression profiles between primary knee osteoarthritis and an osteoarthritis endemic to Northwestern China, Kashin-Beck disease. Arthritis Rheum..

[6] Luo M (2014). Changes in the metabolism of chondroitin sulfate glycosaminoglycans in articular cartilage from patients with Kashin-Beck disease. Osteoarthritis Cartilage.

[7] Wu WH (2017). Comparison of microRNA expression profiles of KashinBeck disease, osteoarthritis and rheumatoid arthritis. Sci. Rep..

[8] Wang W (2017). Genome-wide DNA methylation profiling of articular cartilage reveals significant epigenetic alterations in Kashin-Beck disease and osteoarthritis. Osteoarthritis Cartilage.

[9] Wang S, Guo X, Wang W, Wang S (2012). Genome-wide study identifies the regulatory gene networks and signaling pathways from chondrocyte and peripheral blood monocyte of Kashin-Beck disease. Genes Cells.

[10] Cao J (2008). Articular cartilage metabolism in patients with Kashin-Beck Disease: an endemic osteoarthropathy in China. Osteoarthritis Cartilage.

[11] Wang SJ (2006). Chondrocyte apoptosis and expression of Bcl-2, Bax, Fas, and iNOS in articular cartilage in patients with Kashin-Beck disease. J. Rheumatol..

[12] Ma WJ, Guo X, Yu YX, Gao ZQ (2014). Cytoskeleton remodeling and oxidative stress description in morphologic changes of chondrocyte in Kashin-Beck disease. Ultrastruct. Pathol..

[13] Johnstone B (2013). Tissue engineering for articular cartilage repair - the state of the art. Eur. Cells Mater..

[14] Musumeci G (2014). Advantages of exercise in rehabilitation, treatment and prevention of altered morphological features in knee osteoarthritis. A narrative review. Histol. Histopathol..

[15] Carballo CB, Nakagawa Y, Sekiya I, Rodeo SA (2017). Basic science of articular cartilage. Clin. Sport Med..

[16] Ji QB (2019). Single-cell RNA-seq analysis reveals the progression of human osteoarthritis. Ann. Rheum. Dis..

[17] Jiang YZ, Tuan RS (2015). Origin and function of cartilage stem/progenitor cells in osteoarthritis. Nat. Rev. Rheumatol..

[18] Jeon OH (2017). Local clearance of senescent cells attenuates the development of post-traumatic osteoarthritis and creates a pro-regenerative environment. Nat. Med..

[19] Childs BG (2017). Senescent cells: an emerging target for diseases of ageing. Nat. Rev. Drug Discov..

[20] Koelling S (2009). Migratory chondrogenic progenitor cells from repair tissue during the later stages of human osteoarthritis. Cell Stem Cell.

[21] Saito T (2010). Transcriptional regulation of endochondral ossification by HIF-2 alpha during skeletal growth and osteoarthritis development. Nat. Med..

[22] Maes C, Carmeliet G, Schipani E (2012). Hypoxia-driven pathways in bone development, regeneration and disease. Nat. Rev. Rheumatol..

[23] He Y (2018). 3-morpholinosydnonimine (SIN-1)-induced oxidative stress leads to necrosis in hypertrophic chondrocytes in vitro. Biomed. Pharmacother..

[24] Guo X (2014). Recent advances in the research of an endemic osteochondropathy in China: Kashin-Beck disease. Osteoarthritis Cartilage.

[25] Sun H (2020). Single-cell RNA-seq analysis identifies meniscus progenitors and reveals the progression of meniscus degeneration. Ann. Rheum. Dis..

[26] Valenzi E (2019). Single-cell analysis reveals fibroblast heterogeneity and myofibroblasts in systemic sclerosis-associated interstitial lung disease. Ann. Rheum. Dis..

[27] Ferguson GB (2018). Mapping molecular landmarks of human skeletal ontogeny and pluripotent stem cell-derived articular chondrocytes. Nat. Commun..

[28] Stephenson W (2018). Single-cell RNA-seq of rheumatoid arthritis synovial tissue using low-cost microfluidic instrumentation. Nat. Commun..

[29] Blanco FJ, Valdes AM, Rego-Perez I (2018). Mitochondrial DNA variation and the pathogenesis of osteoarthritis phenotypes. Nat. Rev. Rheumatol..

[30] Blanco FJ, Rego-Perez I (2018). Mitochondria and mitophagy: biosensors for cartilage degradation and osteoarthritis. Osteoarthritis Cartilage.

[31] Liu JT (2010). Mitochondrial function is altered in articular chondrocytes of an endemic osteoarthritis, Kashin-Beck disease. Osteoarthritis Cartilage.

[32] Blanco FJ, Rego I, Ruiz-Romero C (2011). The role of mitochondria in osteoarthritis. Nat. Rev. Rheumatol..

[33] Babula P (2012). Mammalian metallothioneins: properties and functions. Metallomics.

[34] Wu C (2013). Metallothioneins negatively regulate IL-27-induced type 1 regulatory T-cell differentiation. Proc. Natl Acad. Sci. USA.

[35] Wang W (2013). Oxidative stress and status of antioxidant enzymes in children with Kashin-Beck disease. Osteoarthritis Cartilage.

[36] Quintin A (2010). Plasticity of fetal cartilaginous cells. Cell Transplant..

[37] Fickert S, Fiedler J, Brenner RE (2004). Identification of subpopulations with characteristics of mesenchymal progenitor cells from human osteoarthritic cartilage using triple staining for cell surface markers. Arthritis Res. Ther..

[38] Williams R (2010). Identification and clonal characterisation of a progenitor cell sub-population in normal human articular cartilage. PLoS ONE.

[39] Karlsson C, Lindahl A (2009). Articular cartilage stem cell signalling. Arthritis Res Ther..

[40] Hattori S, Oxford C, Reddi AH (2007). Identification of superficial zone articular chondrocyte stem/progenitor cells. Biochem. Biophys. Res. Commun..

[41] Hayes AJ, Tudor D, Nowell MA, Caterson B, Hughes CE (2008). Chondroitin sulfate sulfation motifs as putative biomarkers for isolation of articular cartilage progenitor cells. J. Histochem. Cytochem..

[42] Brack AS, Rando TA (2012). Tissue-specific stem cells: lessons from the skeletal muscle satellite cell. Cell Stem Cell.

[43] Dowthwaite GP (2004). The surface of articular cartilage contains a progenitor cell population. J. Cell Sci..

[44] Alsalameh S, Amin R, Gemba T, Lotz M (2004). Identification of mesenchymal progenitor cells in normal and osteoarthritic human articular cartilage. Arthritis Rheum..

[45] Hiraoka K, Grogan S, Olee T, Lotz M (2006). Mesenchymal progenitor cells in adult human articular cartilage. Biorheology.

[46] Seol D (2012). Chondrogenic progenitor cells respond to cartilage injury. Arthritis Rheum..

[47] Tong WX (2015). In vivo identification and induction of articular cartilage stem cells by inhibiting NF-kappa B signaling in osteoarthritis. Stem Cells.

[48] Kozhemyakina E (2015). Identification of a Prg4-expressing articular cartilage progenitor cell population in mice. Arthritis Rheum..

[49] Yu Y, Zheng H, Buckwalter JA, Martin JA (2014). Single cell sorting identifies progenitor cell population from full thickness bovine articular cartilage. Osteoarthritis Cartilage.

[50] Khan IM, Williams R, Archer CW (2009). One flew over the progenitor’s nest: migratory cells find a home in osteoarthritic cartilage. Cell Stem Cell.

[51] Johnson VL, Hunter DJ (2014). The epidemiology of osteoarthritis. Best Pract. Res. Clin. Rheumatol..

[52] Nevitt MC, Felson DT, Williams EN, Grady D, Replace HEP (2001). The effect of estrogen plus progestin on knee symptoms and related disability in postmenopausal women - The Heart and Estrogen/Progestin Replacement Study, a randomized, double-blind, placebo-controlled trial. Arthritis Rheum..

[53] Wu C (2014). Defective autophagy in chondrocytes with Kashin-Beck disease but higher than osteoarthritis. Osteoarthritis Cartilage.

[54] Zheng J (2013). Abnormal expression of chondroitin sulphate N-acetylgalactosaminyltransferase 1 and Hapln-1 in cartilage with Kashin-Beck disease and primary osteoarthritis. Int. Orthop..

[55] Tong WX (2019). Wnt16 attenuates osteoarthritis progression through a PCP/JNK-mTORC1-PTHrP cascade. Ann. Rheum. Dis..

[56] Yao X (2021). Chondrocyte ferroptosis contribute to the progression of osteoarthritis. J. Orthop. Transl..

[57] Chen T, Hilton MJ, Brown EB, Zuscik MJ, Awad HA (2013). Engineering superficial zone features in tissue engineered cartilage. Biotechnol. Bioeng..

[58] Schumacher BL, Block JA, Schmid TM, Aydelotte MB, Kuettner KE (1994). A novel proteoglycan synthesized and secreted by chondrocytes of the superficial zone of articular-cartilage. Arch. Biochem. Biophys..

[59] Lorenzo P, Bayliss MT, Heinegard D (1998). A novel cartilage protein (CILP) present in the mid-zone of human articular cartilage increases with age. J. Biol. Chem..

