# Supplementary material for: Comparison of the major cell populations among osteoarthritis, Kashin–Beck disease and healthy chondrocytes by single-cell RNA-seq analysis
Source: Cell Death Dis. 2021 May 27;12(6):551. doi: 10.1038/s41419-021-03832-3 (PMC8160352; doi:10.1038/s41419-021-03832-3)
Supplement: Supplementary file 1 — Table S1 [file 41419_2021_3832_MOESM1_ESM.docx]

**Table S1 Characteristics of healthy control, patients from KBD and OA used for Single-cell RNA-seq analysis**

| Sample |  | Age (years) | Gender | BMI | TKA | Grade^*^ |
| --- | --- | --- | --- | --- | --- | --- |
| Healthy control |  | 61 | Female  Female  Female | 21.3 | Left | - |
| KBD |  | 60 |  | 24.5 | Left | Ⅲ |
| OA |  | 49 |  | 22.5 | Left | Ⅲ |

* grade Ⅲ KBD patient according to the national diagnostic criteria of KBD in China [WS/T 207-2010]; grade Ⅲ OA patients according to the Kellgren Lawrence scoring system. TKA: total knee arthroplasty
