# Supplementary material for: Comparison of the major cell populations among osteoarthritis, Kashin–Beck disease and healthy chondrocytes by single-cell RNA-seq analysis
Source: Cell Death Dis. 2021 May 27;12(6):551. doi: 10.1038/s41419-021-03832-3 (PMC8160352; doi:10.1038/s41419-021-03832-3)
Supplement: Supplementary file 2 — Table S2 [file 41419_2021_3832_MOESM2_ESM.docx]

| Sample |  | Age (years) | Gender |  | BMI | | TKA | Grade | | |
| --- | --- | --- | --- | --- | --- | --- | --- | --- | --- | --- |
| IHC  Normal 1 |  | 30 | Female  Female  Female  Male  Male | 23.1 | | Right  Right  Left  Right  Left | | | - |  |
| Normal 2 |  | 44 |  | 22.7 | |  |  |  | - |  |
| Normal 3 |  | 55 |  | 21.8 | |  |  |  | - |  |
| Normal 4 |  | 42 |  | 22.9 | |  |  |  | - |  |
| Normal 5 |  | 49 |  | 19 | |  |  |  | - |  |
| KBD 1 |  | 77 | Female | 26.7 | | Left | | | Ⅱ^*^ |  |
| KBD 2 |  | 62 | Female | 22.6 | | Right | | | Ⅲ^*^ |  |
| KBD 3 |  | 54 | Female | 24.7 | | Left | | | Ⅲ^*^ |  |
| KBD 4 |  | 56 | Male | 21.3 | | Right | | | Ⅲ^*^ |  |
| KBD 5 |  | 66 | Male | 22.5 | | Left | | | Ⅱ^*^ |  |
| OA 1 |  | 74 | Female | 25.8 | | Right | | | Ⅲ^*^ |  |
| OA 2 |  | 69 | Female | 23.3 | | Left | | | Ⅲ^*^ |  |
| OA 3 |  | 47 | Female | 19.5 | | Left | | | Ⅲ^*^ |  |
| OA 4 |  | 63 | Male | 24 | | Right | | | Ⅲ^*^ |  |
| OA 5  qRT-PCR  Normal 1  Normal 2  Normal 3 |  | 62  35  39  45 | Male  Female  Male  Male | 24.2  25.1  20.4  22.7 | | Left  Left  Left  Right | | | Ⅲ^*^  -  -  - |  |
| KBD 1 |  | 69 | Female | 26.6 | | Left | | | Ⅲ^*^ |  |
| KBD 2 |  | 73 | Male | 21.7 | | Right | | | Ⅲ^*^ |  |
| KBD 3 |  | 58 | Female | 20.1 | | Left | | | Ⅱ^*^ |  |
| OA 1 |  | 68 | Female | 23.4 | | Left | | | Ⅲ^*^ |  |
| OA 2 |  | 77 | Male | 19.5 | | Left | | | Ⅲ^*^ |  |
| OA 3 |  | 66 | Male | 22.5 | | Right | | | Ⅲ^*^ |  |

**Table S2 Characteristics of healthy control, patients from KBD and OA used for IHC and qRT-PCR analysis**

* grade Ⅱ and Ⅲ KBD patient according to the national diagnostic criteria of KBD in China [WS/T 207-2010]; grade Ⅲ OA patients according to the Kellgren Lawrence scoring system. TKA: total knee arthroplasty
