# Supplementary material for: Comparison of the major cell populations among osteoarthritis, Kashin–Beck disease and healthy chondrocytes by single-cell RNA-seq analysis
Source: Cell Death Dis. 2021 May 27;12(6):551. doi: 10.1038/s41419-021-03832-3 (PMC8160352; doi:10.1038/s41419-021-03832-3)
Supplement: Supplementary file 3 — Supplementary figure legends [file 41419_2021_3832_MOESM3_ESM.docx]

**Online supplementary figure legends**

Figure S1 Monocle pseudotime trajectory showing the progression of healthy control chondrocytes. The expression of the genes in a branch-dependent manner. Each row indicates the standardised kinetic curves of a gene. The centre of the heatmap shows the kinetic curve value at the root of the trajectory. From the centre to the left of the heatmap, the kinetic curve progresses from the root along the trajectory to fate 1. Starting from the right, the curve from the root to fate 2.

Figure S2 Violin plots of gene expression of chondrocyte markers in different cell clusters with cells separated by disease status.

Figure S3 The verification of the gene expressions of MT-ND1 and MT1X in patients with KBD (n=3) using RT-PCR. The verification of the gene expressions of AEBP1 and CHI3L1 in patients with OA (n=3) using RT-PCR. **, P<0.01

Figure S4 Representative immunohistochemistry staining of PTTG1 and BIRC5 in OA and KBD tissues. Scale bar, left, 500 μm; right, 50 μm, and comparative quantification of positive cells of different areas (superficial, middle, deep) between OA and KBD tissues displayed by box plot (n=5). *p<0.05

Figure S5 Single-cell RNA-sequencing analysis of KBD chondrocyte tissue samples. (A) Seven KBD chondrocyte clusters. Visualisation of clustering by t-SNE plot of KBD samples, identified by cell type. (B) Violin plots of gene expression of chondrocyte markers in ECs (C2orf82, MIA, S100B and FRZB) and HTCs (IBSP, MMP13, RUNX2 and TMEM119) populations.

Figure S6 Single-cell RNA-sequencing analysis of OA chondrocyte tissue samples. (A) Seven OA chondrocyte clusters. Visualisation of clustering by t-SNE plot of OA samples, identified by cell type. (B) Violin plots of gene expression of chondrocyte markers in ECs (MIA, S100B, S100A1 and FRZB) populations.
