# Supplementary figures and images for: Comparison of the major cell populations among osteoarthritis, Kashin–Beck disease and healthy chondrocytes by single-cell RNA-seq analysis
Source: Cell Death Dis. 2021 May 27;12(6):551. doi: 10.1038/s41419-021-03832-3 (PMC8160352; doi:10.1038/s41419-021-03832-3)

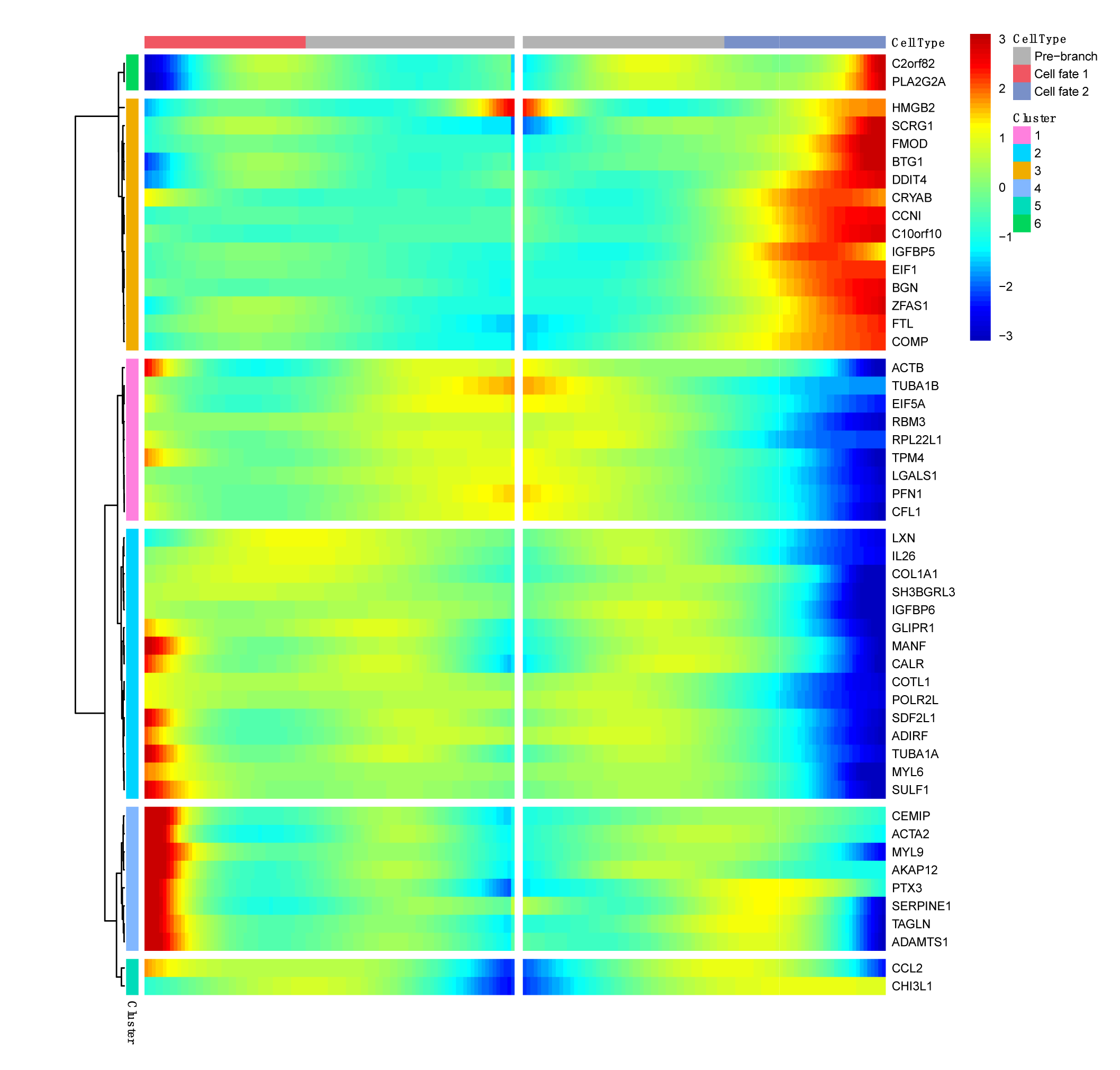

Supplement: Supplementary file 4 — online supplementary figure 1 [file 41419_2021_3832_MOESM4_ESM.tif]

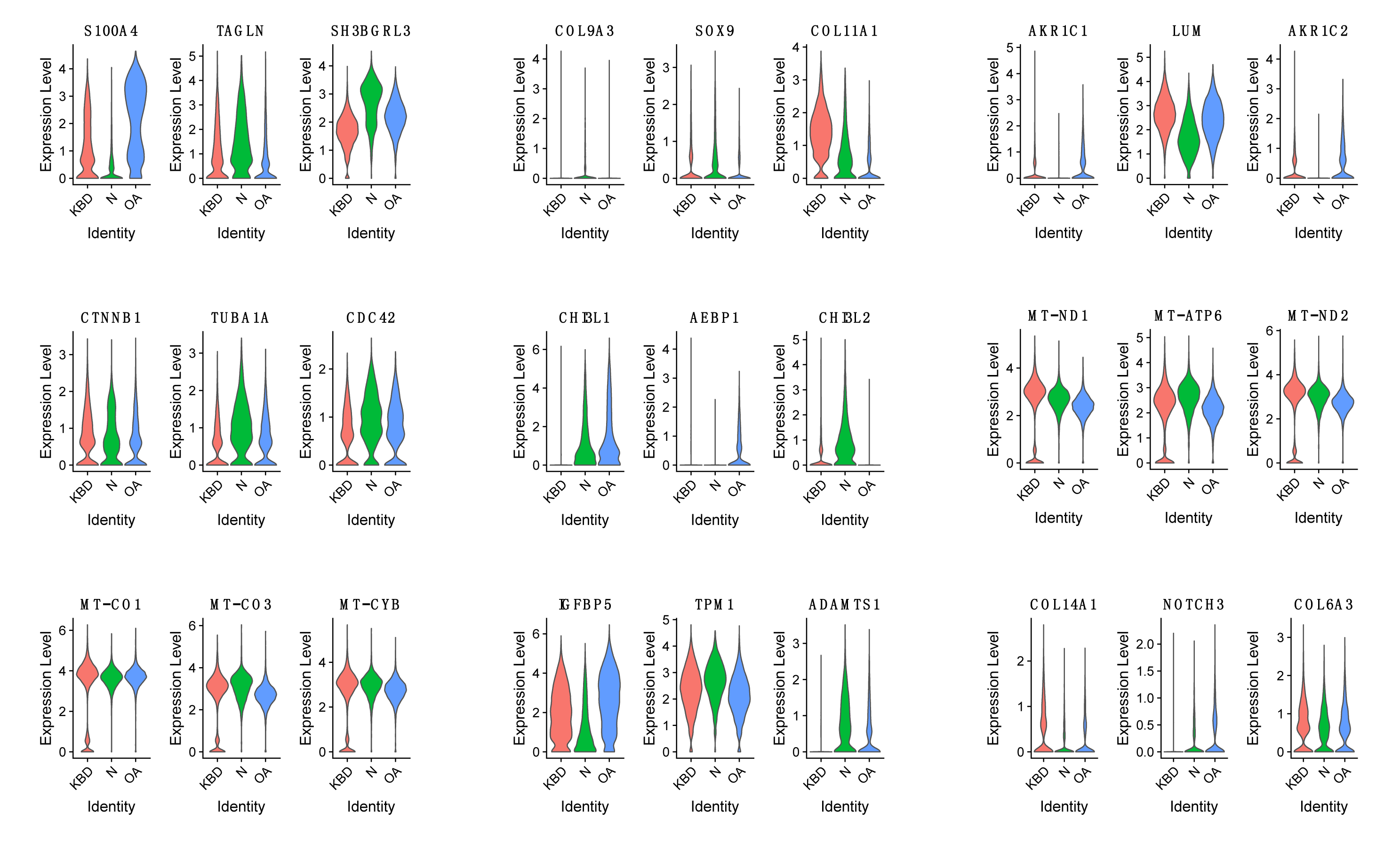

Supplement: Supplementary file 5 — online supplementary figure 2 [file 41419_2021_3832_MOESM5_ESM.tif]

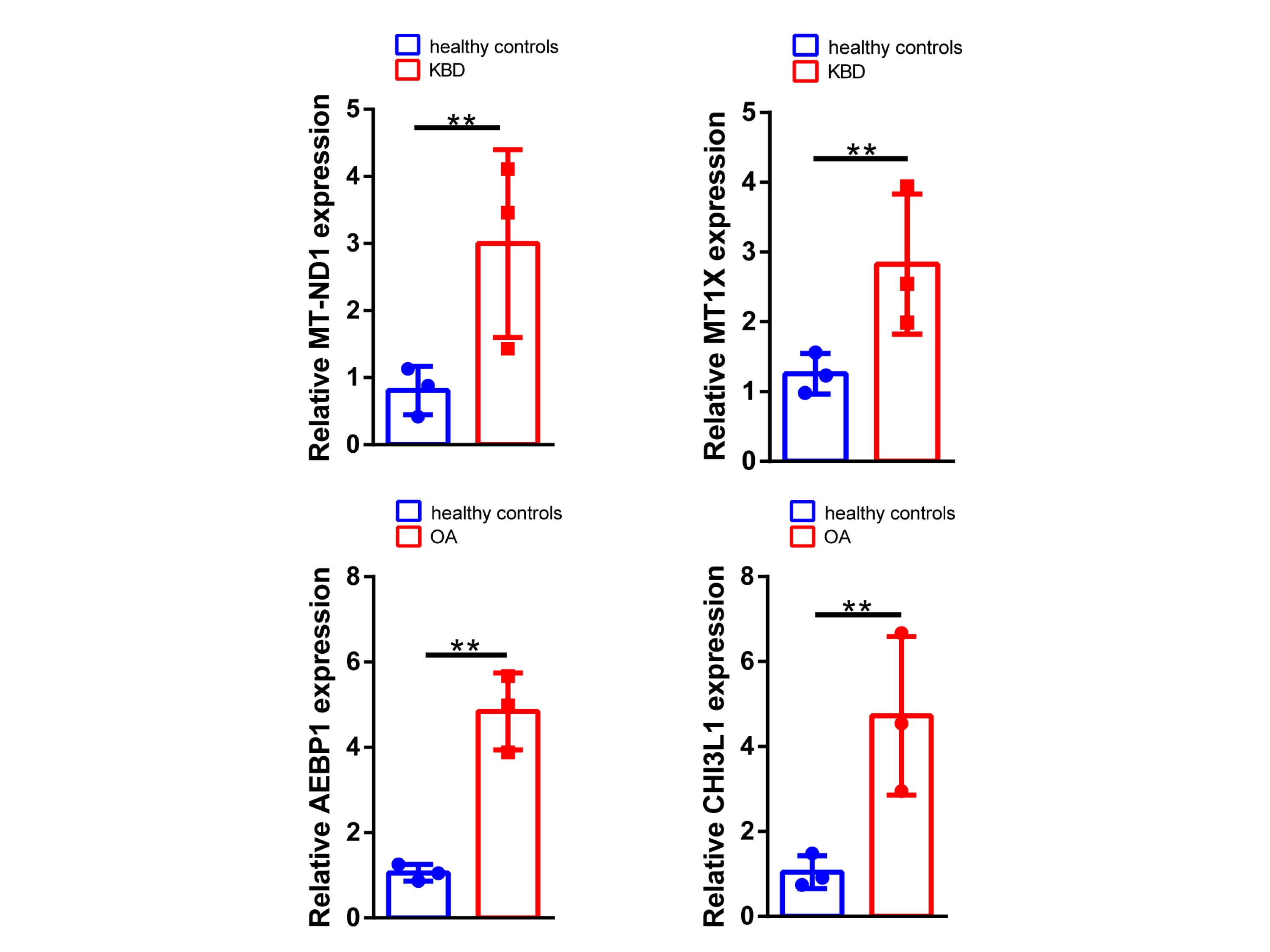

Supplement: Supplementary file 6 — online supplementary figure 3 [file 41419_2021_3832_MOESM6_ESM.tif]

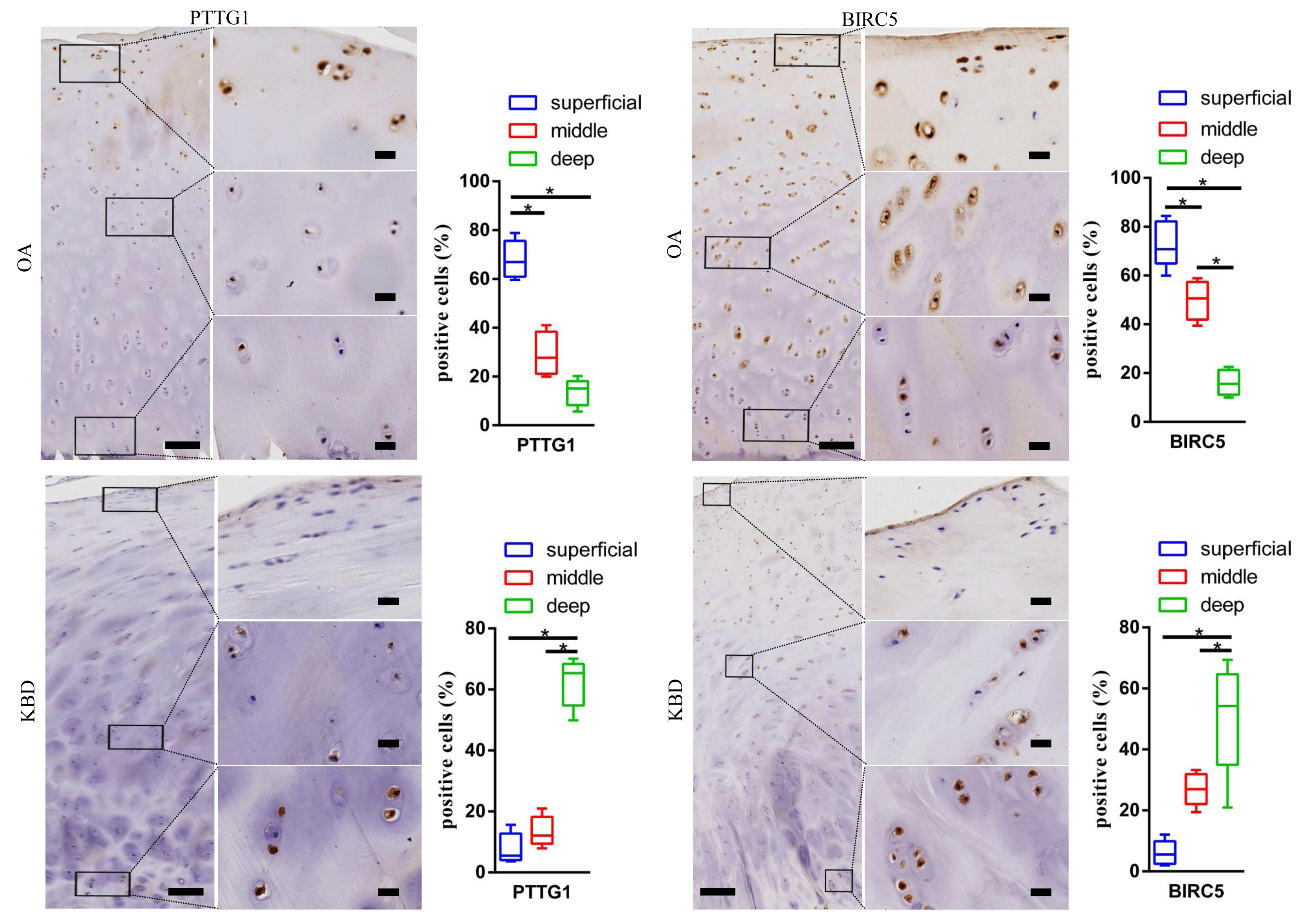

Supplement: Supplementary file 7 — online supplementary figure 4 [file 41419_2021_3832_MOESM7_ESM.tif]

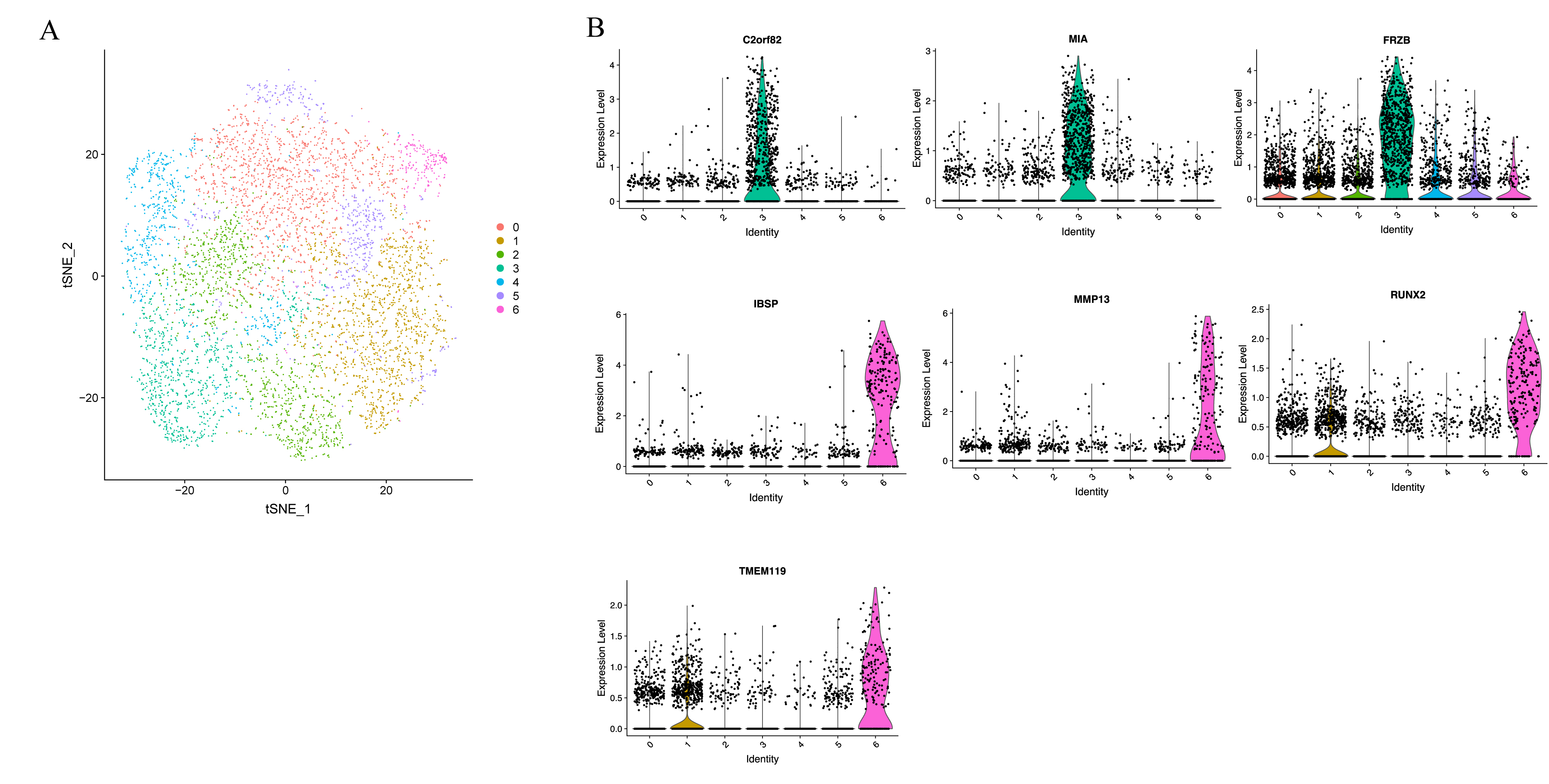

Supplement: Supplementary file 8 — online supplementary figure 5 [file 41419_2021_3832_MOESM8_ESM.tif]

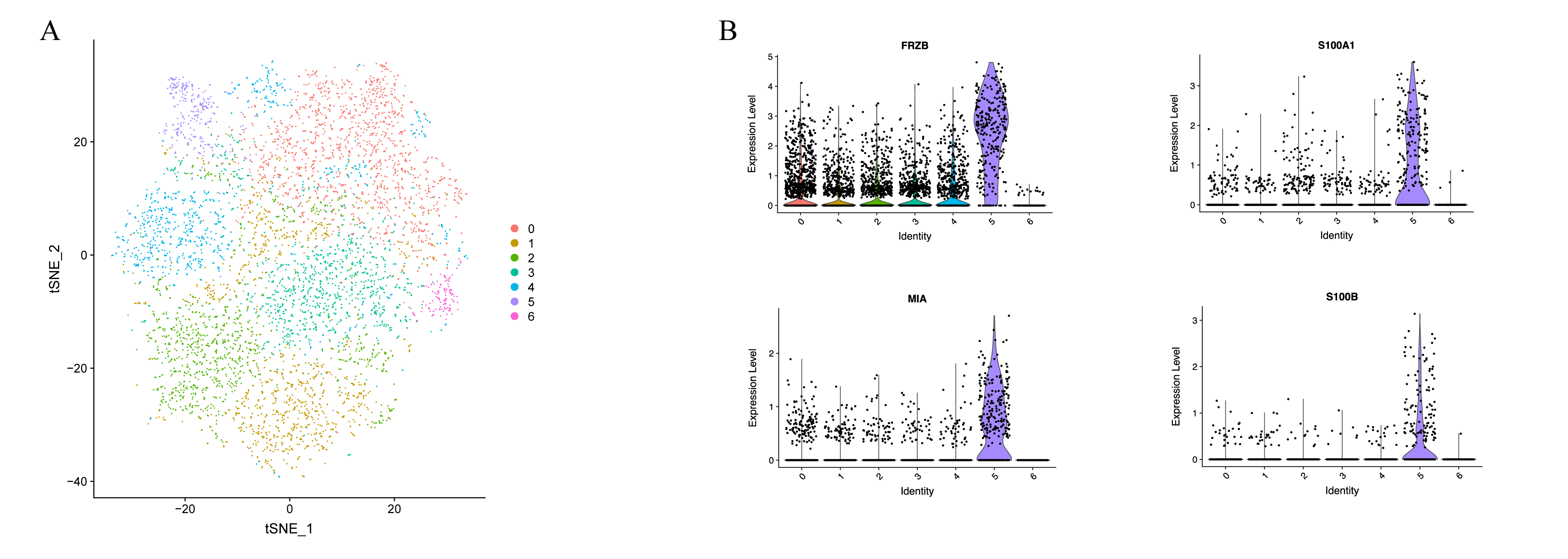

Supplement: Supplementary file 9 — online supplementary figure 6 [file 41419_2021_3832_MOESM9_ESM.tif]
